# No apparent p53 activation in CRISPR‐engineered gene‐edited rabbits

**DOI:** 10.1111/jcmm.16960

**Published:** 2021-10-05

**Authors:** Tao Zhang, JinZe Li, Tian Wang, Feiyu Zhao, Tingting Sui

**Affiliations:** ^1^ Institute of Zoonosis Jilin University Changchun China

**Keywords:** BEs, CRISPR‐Cas9, gene editing, p53, rabbit

## Abstract

Clustered regularly interspaced short palindromic repeats‐CRISPR‐associated 9 (CRISPR‐Cas9) and base editors (BEs) are revolutionary gene‐editing technology that has been widely utilized in biology, biotechnology and medicine. However, recent reports show that CRISPR‐Cas9‐mediated genome editing can induce a p53‐mediated stress response and cell cycle arrest in human cells, while not illustrated in gene‐editing animals. In the study, to verify whether there is a phenomenon of p53 activation, by analysing nine gene‐edited rabbits using CRISPR‐Cas9 and BEs, we provide the first evidence that no apparent p53 expression changes in those rabbits generated by Cas9 or BE‐edited, suggesting that p53 may not need to consider for application in gene‐edited animals.

## 1 INTRODUCTION

Clustered regularly interspaced short palindromic repeats‐CRISPR‐associated 9 (CRISPR‐Cas9) involves the generation of DNA double‐strand breaks (DSB) induced by the RNA‐guided Cas9 endonuclease,^1^ repaired by either non‐homologous end joining (NHEJ) or homology‐directed repair (HDR). In contrast, the base editors (BEs), that conversion of one base pair to another at a target genomic locus, without requiring DSBs, HDR processes or donor DNA templates.^2^

Tumour protein 53 (*TP53*) is a potent tumour suppressor mutated in many human cancers, which regulates cellular metabolism, apoptosis and DNA damage repair.^3^ Thus, it should come as no surprise that p53 is a key player in CRISPR/Cas9‐based genome editing. Indeed, recent studies have shown that CRISPR‐Cas9‐mediated genome editing in normal cells triggered a p53‐mediated DNA damage response,^4^, ^5^ which induces DNA damage response (DDR) and activates the expression of downstream effector proteins, such as cell cycle inhibitor p21. A single DSB induced by CRISPR‐Cas9 leads to p53‐dependent cellular toxicity, and cell‐cycle arrest in human cells and significantly hamper the efficiency of precise genomic editing, which could cause potential problems, especially the efficacy and safety for direct in vivo gene‐editing applications. Therefore, it will be important to examine the tumourigenic potential of ex vivo CRISPR‐edited cells in animal models and the consequences of concurrent p53 loss.

To investigate whether CRISPR‐Cas9 or base editing caused p53‐induced changes *in vitro*, the 9 lines of gene‐edited rabbits were used for analysis. Here, we present the first evidence that there were no apparent p53 expression changes in rabbits generated by CRISPR‐Cas9 or BEs system.

## 2 MATERIALS AND METHODS

### 2.1 Animal and ethics statement

The gene‐editing rabbits were generated in our laboratory; the *DMD*, *XIST*, *DMP1*, *FAM83h*, *FBN1* and *CD300LF* gene editing rabbits were generated by CRISPR/Cas9 system; the *TYR*, *FUT1* and *OTC* gene editing rabbits were generated by BEs system. All protocols using rabbits were approved and performed with the guidelines of the Animal Care and Use Committee of Jilin University.

### 2.2 Genotyping of the gene‐editing rabbits

Genomic DNA was extracted from a small piece of ear tissue of CRISPR‐edited rabbits using a TIANamp Genomic DNA Kit (Tiangen) according to the manufacturer's instructions. The PCR amplification primers are listed in Table S1.

### 2.3 Western blotting assay

Western blotting was performed based on the previously described protocol.^6^ Briefly, tissues were lysed with RIPA buffer supplemented with protease inhibitor (PI, Thermo Scientific) and phenylmethanesulfonyl fluoride (PMSF, Roche Applied Science) on ice for 30 min and vortex briefly evert 10 min. Protein concentrations were measured with BCA Protein Assay Kit (Beyotime). First of all, a total of 40 ug of protein from each tissue sample was separated by 12% SDS‐PAGE and then electrophoretically transferred to a polyvinylidene difluoride membrane (PVDF). Next, blocked the membranes with 3% BSA/Tris‐buffered saline/Tween (TBS‐T) for 1 h at room temperature, then incubated with anti‐p53 monoclonal antibody (1:2000, Proteintech 60283–2) and anti‐Beta actin monoclonal antibody (1:5000, Proteintech 60008–1) overnight at 4°C, and incubated with HRP‐anti‐mouse for 60 min at room temperature. Finally, bands were visualized by enhanced chemiluminescence solution (ECL, Meilun). Image‐J was used to quantify the band signals.

### 2.4 Immunofluorescence staining

An immunofluorescence assay was performed as previously described.^7^ The paraffin‐embedded tissues were deparaffinized and antigen retrieval was performed using microwave oven heating. After blocking with 10% goat serum in PBS for 1 h at room temperature and incubated with an antibody against p53 (diluted in PBS with 1% BSA) at 4°C overnight (60283–2, Proteintech) was used. Then, the tissues were incubated for 1 h at room temperature with secondary antibodies (Alexa Fluor 555‐conjugated goat anti‐mouse IgG, Cell Signaling Technology). Finally, the nuclei were stained with 4’,6‐diamidino‐2‐phenylindole (DAPI) (Sigma‐Aldrich). The coverslips were then sealed with glycerol and images were captured with a laser confocal microscopy (Fluoview FV1200, Olympus). Image‐Pro Plus software was used (Media Cybernetics, Rockville MD) to quantify the immunofluorescent signals.

### 2.5 Statistical analysis

Data were statistically analysed with GraphPad Prism software (*t*‐test) and expressed as mean ±standard error of mean (SEM), and *p* <  0.05 was considered statistically significant.

## 3 RESULTS

To analysis whether changes of p53‐induced in vitro editing by CRISPR‐Cas9 or BE, nine mutant rabbit lines generated using CRISPR‐Cas9 or BEs were utilized in this study (Table 1). We grouped these rabbits as follows: (1) the genome editing rabbits generated with DSB by CRISPR‐Cas9; (2) the genome editing rabbit generated without DSB by BEs. Genotypes were determined by PCR (Figure S1).

**TABLE 1:** Summary of the CRISPR induced a p53 expression changes in rabbits

Gene name	Rabbits lines	Nature of mutation	DSB	P53 changes	Method
CD300LF	C1 (Figure S2)	−157/−70bp in exon 51	YES	No	CRISPR/Cas9
XIST	X1 (Figure S4)	−193bp in exon 1	YES	No	CRISPR/Cas9
FAM83h	F1 (Figure S5)	C>T in exon 14	YES	No	CRISPR/Cas9
DMD	D1 (Figure S6)	−157/−70bp in exon 51	YES	No	CRISPR/Cas9
FBN1	N1 (Figure S7)	−7/−4bp in exon 65	YES	No	CRISPR/Cas9
DMP1	P1 (Figure S8)	−522bp in exon 1 and 2	YES	No	CRISPR/Cas9
TYR	T1 (Figure S3)	C>T in exon 1	NO	NO	BE3
FUT1	U1 (Figure S9)	C>T in exon 1	NO	NO	BE3
OTC	O1 (Figure S10)	A>G in exon 1	NO	NO	ABE

### 3.1 p53 expression analysis of the rabbits generated with DSB by CRISPR‐Cas9

**FIGURE 1:**
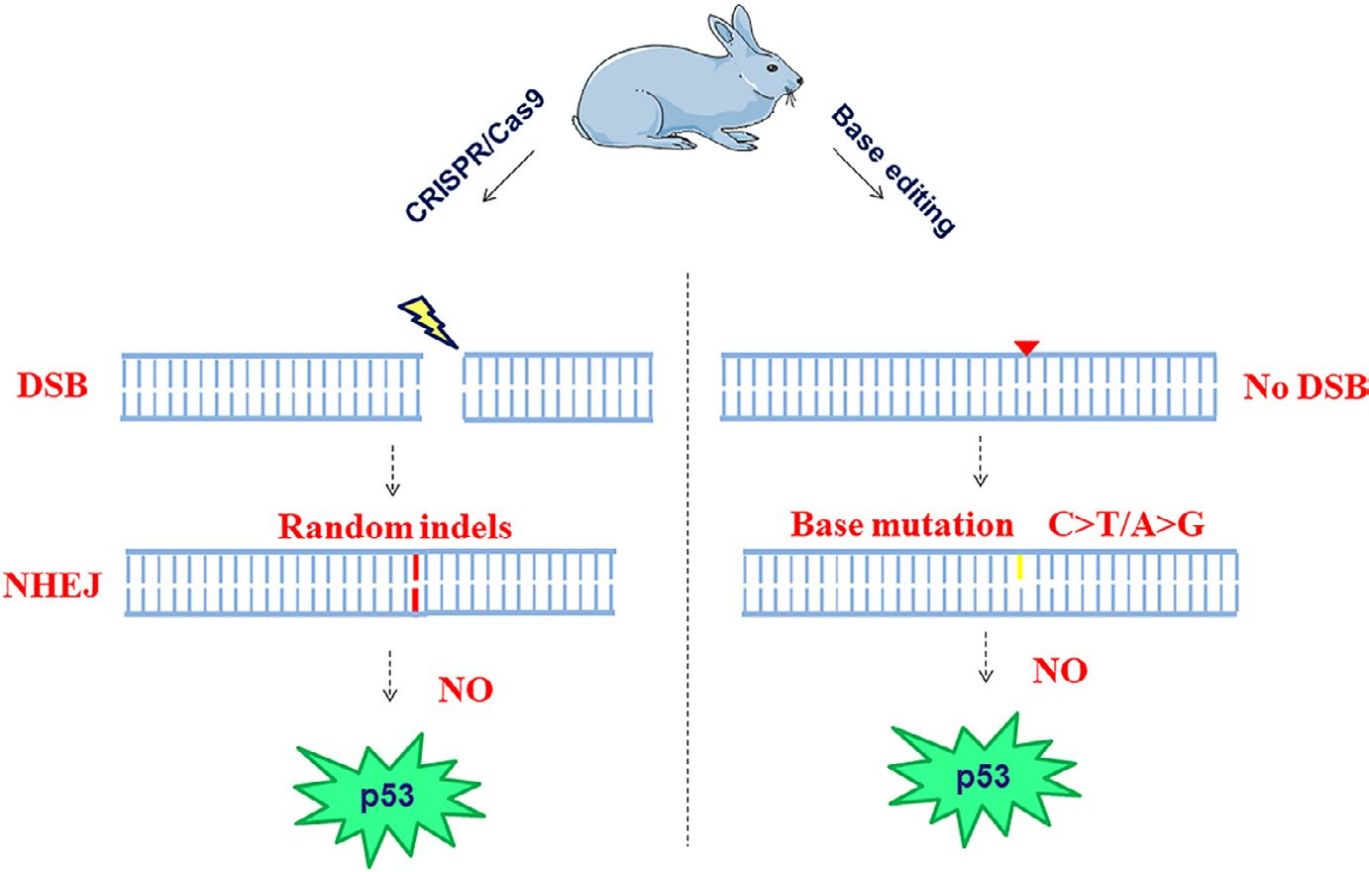
Expression of p53 not changes in rabbits generated by Cas9 or BE‐edited. When CRISPR/Cas9 make a single DSB in chromosome, inaccurately repair via nonhomologous end joining (NHEJ), this is the normal genome‐editing outcome, which not induces the expression of p53 changes in rabbits. On the contrary, the BEs‐mediated gene‐editing is not generated the DSB, it only provides precise editing at a single base‐pair conversion not causes the p53 changes

It has been shown that CRISPR‐Cas9‐mediated genetic modification in normal cultured cells derived a p53‐dependent toxic,^4^, ^5^ which may raise tumour risks. To determine whether gene‐edited rabbits may be exposed to the same risk, the designed targeting sites of C1(Figure S2A), X1(Figure S4A), F1(Figure S5A), D1(Figure S6A), N1(Figure S7A) and P1(Figure S8A) rabbits were showed and generated by CRISPR‐Cas9 were analysed (Table 1). There was no significant change in the protein expression of p53 in lung and liver between C1 gene‐edited group and WT rabbits (Figure S2B,C). By Western blot analysis, other lines of gene‐edited rabbits also showed no changes in p53 expression compared with WT controls (Figure S4B,C; Figure S5B,C; Figure S6B,C; Figure S7B,C; Figure S8B,C). In order to further verify the p53 expression, the immunofluorescence staining was performed. Analysis of the results remained no apparent changes of p53 expression in edited rabbits compared with WT controls (Figure S2D,E Figure S4D,E; Figure S5D,E; Figure S6D,E; Figure S7D,E; Figure S8D,E). These data suggest that CRISPR‐Cas9‐mediated genome editing did not induce apparent p53 expression changes in rabbit tissues, which is consistent with the previous study ^8^ (Figure 1).

### 3.2 p53 expression analysis of the rabbits generated by BEs

Currently, base editors that provide precise editing at a single base‐pair, without generating DSBs have been widely reported in recent study.^9^ Thus, we tried to verify and replicate these results in BEs‐mediated genome editing rabbits, using T1, U1 and O1 individuals (Table 1), the targeting sites are shown in Figures S3A,9A,10A. The western blotting and immunofluorescence staining were performed to detect the expression of p53. The skin and heart of T1 gene editing rabbits exhibited no apparent changes of p53 expression compared with WT control (Figure S3B,C). In addition, the results indicated that there were no apparent changes of p53 expression between more edited rabbits and WT controls by western blotting assay (Figure S9B,C; Figure S10B,C) and the immunofluorescence staining (Figure S3D,E; Figure S9D,E; Figure S10D,E), suggesting that BEs‐mediated base editing did not affect p53 expression in rabbits using the BEs system (Figure 1).

Furthermore, in order to examine tumourigenic potential of ex vivo CRISPR‐edited cells in rabbits, 26 lines of gene editing rabbits in our laboratory were used to observe the health condition, the results showed that these gene‐edited rabbits displayed a phenotype with autologous gene mutations, so far, no tumours have been found in the development of gene‐edited rabbits, so it is sufficient to show that the gene‐edited rabbits are healthy without tumour susceptibility (Table S2).

## 4 DISCUSSION

In this study, a large number of CRISPR‐edited rabbits were used to examine the tumourigenic potential and confirmed that there are no p53 expression changes, which is consistent with previous studies.^8^, ^10^ Thus, we think the CRISPR/Cas9 or BE‐mediated genome editing may be safe in clinical applications.

CRISPR‐Cas9‐mediated genome editing involves the generation of DSBs, which is repaired by NHEJ and HDR.^11^ It is evident that DNA repair process may cause unexpected results, including precise genome editing, chromatin rearrangements and insertions or deletions (Indels), and off‐target. TP53, as a potent tumour suppressor that is the most potent cell cycle checkpoint by cell cycle arrest in response to DNA damage.^3^ Although a series of studies have described severe deleterious consequences of CRISPR‐Cas9‐induced DNA damage, these cases were related to DSBs.^12^ It is surprising that a single DSB is enough to cause a prolonged p53‐dependent cell death and affect the efficiency of precise genomic editing. Furthermore, temporary inhibition of TP53 may increase the genome editing efficiency of primary and TP53^+/+^ cell lines.^13^ However, this phenomenon was just reported in non‐transformed retinal pigmented epithelial cells,^5^ embryonic stem cells (hESCs) and induced pluripotent stem cells (iPSCs).^4^ In our study, DSBs were generated in the C1, X1, D1, N1, F1 and P1 rabbits with no apparent changes in p53 expression. These results are not consistent with previous report that CRISPR‐Cas9‐induced DSBs triggered a p53‐dependent toxic response and caused a growth arrest in human cells.^4^ CRISPR‐Cas9‐induced DNA damage may lead to p53 activation, but molecular mechanism leading to response and that mitigates this response in the absence of genetic selection to allow Cas9 tolerance remain to be elucidated.^14^ We speculate that Cas9‐induced p53 activation is only manifested during embryonic development, and embryonic development, some embryos that cause p53 overactivation due to Cas9 induction may die during development in animals, which is different from cells.

In addition, another important problem is that the emerging non‐DSB inducing and relying on cellular mismatch repair mechanism, such as BEs, only provide precise editing at a single base‐pair conversion whether also induce DSB and p53 activity. The BEs contain a UGI motif to prevent activation of DNA damage response machinery.^15^ In this study, we found that the BEs‐mediated modification did not trigger p53 expression in base editing rabbits (T1, U1 and O1 ). If p53 is induced by DSB, the BEs‐mediated genome editing may represent a viable alternative for basic research and screening approaches.

## 5 CONCLUSIONS

Overall, this study is the first detailed report that no apparent changes of p53 expression in detected tissues of rabbits by CRISPR‐Cas9 or BEs editing. These findings further highlight that there is no p53 activation in CRISPR gene‐edited rabbits.

## CONFLICT OF INTEREST

The authors confirm that there are no conflicts of interest.

## AUTHOR CONTRIBUTION

**Tingting Sui:** Conceptualization (equal); Writing‐review & editing (equal). **Tao Zhang:** Conceptualization (equal); Writing‐original draft (equal). **Jinze Li:** Formal analysis (equal); Validation (equal). **Tian Wang:** Writing‐review & editing (equal). **Feiyu zhao:** Data curation (equal); Supervision (equal).

## Supporting information

Supplementary Material

## Data Availability

The data that support the findings of this study are available from the corresponding author upon reasonable request.
