# Supplementary material for: No apparent p53 activation in CRISPR‐engineered gene‐edited rabbits
Source: J Cell Mol Med. 2021 Oct 5;25(21):10313–7. doi: 10.1111/jcmm.16960 (PMC8572782; doi:10.1111/jcmm.16960)
Supplement: Supplementary file 1 — Supplementary Material [file JCMM-25-10313-s001.docx]

**Supplementary Table**

| Name | | Primers | Sequence (5’-3’) | Produce size (bp) |
| --- | --- | --- | --- | --- |
| DMD  XIST  DMP1  FAM83h  FBN1  CD300LF  TYR  OTC  FUT1 | DMD-F  DMD-R  XIST-F  XIST-R  DMP1-F  DMP1-R  FAM83h-F  FAM83h-R  FBN1-F  FBN1-R  CD300LF-F  CD300LF-R  TYR-F  TYR-R  OTC-F  OTC-R  FUT1-F  FUT1-R | | GTCAACTATCTACTGCAAGAGC  CTGTACTTCATCCCACTGATTC  GTCCTTGGAAGATGGCTCTAAC  AGGAGGAGGTGTCAAGAAGATA  GATAGAGACACACTCCATTGAA  AGCCCTTAACAGTTTCAACA  GAGCTGCTGGAGAAGTACAA  CAGGATCTTGGGCATGAACT  TGCCTCACATCTAGCTCCCT  AGAAACTCCAGAAAGCCCCG  GTGATATTTGCCCTGCTCTT  TGCTCTGATCGTCTCTGAT  ACTACGAGCCCAGACTATGT  GCTCTGTCGGCTATTGTACTC  CCTGCCCGGTAGTATCTCTAA  TTGTGGCTGCATTCCTCTAC  CCTGTGGCTCCCTCATTC  AAGCAGCTGTTTCCACGA | 459  651  862  1194  745  442  432  335  559 |

**Table S1. PCR primers for genotyping of the gene editing rabbits**

**Table S2. Summary of genetically modified rabbits in our laboratory using CRISPR-mediated genome editing**

| **Genes** | **Method** | **Modification** | **Phenotype** | **Tumorigenesis** | **Reference** |
| --- | --- | --- | --- | --- | --- |
| GJA8 | CRISPR/ Cas9 | KO | Congenital cataracts | NO | **^[^**[**^1^**](#_ENREF_1)**^]^** |
| CRYAA | CRISPR/ Cas9 | KO | Congenital cataracts | NO | **^[^**[**^2^**](#_ENREF_2)**^]^** |
| PHEX | CRISPR/ Cas9 | KO | X-linked hypophosphatemia | NO | **^[^**[**^3^**](#_ENREF_3)**^]^** |
| DMD | CRISPR/ Cas9 | KO | Duchenne muscular dystrophy | NO | **^[^**[**^4^**](#_ENREF_4)**^]^** |
| ANO5 | CRISPR/ Cas9 | KO | muscular dystrophy | NO | **^[^**[**^5^**](#_ENREF_5)**^]^** |
| TYR | CRISPR/ Cas9 | KO,large fragment deletion | Coat color | NO | **^[^**[**^6^**](#_ENREF_6)**^]^** |
| MSTN | CRISPR/ Cas9 | KO | Muscle hypertrophy | NO | **^[^**[**^7^**](#_ENREF_7)**^]^** |
| FBN1 | CRISPR/ Cas9 | KO | Marfanoid-progeroid  lipodystrophy syndrome | NO | **^[^**[**^8^**](#_ENREF_8)**^]^** |
| SRY | CRISPR/ Cas9 | KO | Sex reversal syndromes | NO | **^[^**[**^9^**](#_ENREF_9)**^]^** |
| LMNA | CRISPR/ Cas9 | KO | Premature aging syndrome | NO | **^[^**[**^10^**](#_ENREF_10)**^]^** |
| FUT1/FUT2/SEC1 | CRISPR/ Cas9 | KO | Fucosyltransferases enzymes  activity | NO | **^[^**[**^11^**](#_ENREF_11)**^]^** |
| HOXC13 | CRISPR/ Cas9 | KO | hypotrichosis and nail dystrophy | NO | **^[^**[**^12^**](#_ENREF_12)**^]^** |
| XIST | CRISPR/ Cas9 | KO | X chromosome inactivation | NO | **^[^**[**^13^**](#_ENREF_13)**^]^** |
| DMP1 | CRISPR/ Cas9 | KO | hypophosphatemic rickets | NO | **^[^**[**^14^**](#_ENREF_14)**^]^** |
| GCK | CRISPR/ Cas9 | KO | maturity-onset diabetes of the young 2 | NO | **^[^**[**^15^**](#_ENREF_15)**^]^** |
| FGF5 | CRISPR/ Cas9 | KO | Systemic Long Hair | NO | **^[^**[**^16^**](#_ENREF_16)**^]^** |
| OTC | Base editor | Base editing | Ornithine CarbamoyltransferaseDeficiency | NO | **^[^**[**^17^**](#_ENREF_17)**^]^** |
| FGF5 | Base editor | Base editing | Systemic Long Hair | NO | **^[^**[**^17^**](#_ENREF_17)**^]^** |
| TYR | Base editor | Base editing | Coat color | NO | **^[^**[**^18^**](#_ENREF_18)**^]^**  **^[^**[**^18^**](#_ENREF_18)**^]^**  **^[^**[**^18^**](#_ENREF_18)**^]^**  **^[^**[**^18^**](#_ENREF_18)**^]^** |
| MSTN | Base editor | Base editing | Muscle hypertrophy | NO |  |
| LMNA | Base editor | Base editing | Hutchinson-Gilford progeria  Syndrome | NO |  |
| DMD | Base editor | Base editing | Duchenne muscular dystrophy | NO |  |
| CD300LF | CRISPR/ Cas9 | Base editing | murine norovirus receptor | NO | unpublished data  unpublished data  unpublished data  unpublished data |
| APOE | Base editor | Base editing | Lipid metabolism and  atherosclerosis | NO |  |
| LEPTIN | Base editor | Base editing | obesity | NO |  |
| CCR5 | Base editor | Base editing | C-C chemokine receptor 5 | NO |  |

KO:knockout;NO: no find tumor

**Reference**

1. Yuan L, Sui T, Chen M, Deng J, Huang Y, Zeng J, Lv Q, Song Y, Li Z, Lai L. CRISPR/Cas9-mediated GJA8 knockout in rabbits recapitulates human congenital cataracts. Scientific reports 2016, **6:** 22024.

2. Yuan L, Yao H, Xu Y, Chen M, Deng J, Song Y, Sui T, Wang Y, Huang Y, Li Z, Lai L. CRISPR/Cas9-Mediated Mutation of alphaA-Crystallin Gene Induces Congenital Cataracts in Rabbits. Investigative ophthalmology & visual science 2017, **58**(6)**:** BIO34-BIO41.

3. Sui T, Yuan L, Liu H, Chen M, Deng J, Wang Y, Li Z, Lai L. CRISPR/Cas9-mediated mutation of PHEX in rabbit recapitulates human X-linked hypophosphatemia (XLH). Human molecular genetics 2016, **25**(13)**:** 2661-2671.

4. Sui T, Lau YS, Liu D, Liu T, Xu L, Gao Y, Lai L, Li Z, Han R. A novel rabbit model of Duchenne muscular dystrophy generated by CRISPR/Cas9. Disease models & mechanisms 2018, **11**(6).

5. Sui T, Xu L, Lau YS, Liu D, Liu T, Gao Y, Lai L, Han R, Li Z. Development of muscular dystrophy in a CRISPR-engineered mutant rabbit model with frame-disrupting ANO5 mutations. Cell death & disease 2018, **9**(6)**:** 609.

6. Song Y, Zhang Y, Chen M, Deng J, Sui T, Lai L, Li Z. Functional validation of the albinism-associated tyrosinase T373K SNP by CRISPR/Cas9-mediated homology-directed repair (HDR) in rabbits. EBioMedicine 2018, **36:** 517-525.

7. Lv Q, Yuan L, Deng J, Chen M, Wang Y, Zeng J, Li Z, Lai L. Efficient Generation of Myostatin Gene Mutated Rabbit by CRISPR/Cas9. Scientific reports 2016, **6:** 25029.

8. Chen M, Yao B, Yang Q, Deng J, Song Y, Sui T, Zhou L, Yao H, Xu Y, Ouyang H, Pang D, Li Z, Lai L. Truncated C-terminus of fibrillin-1 induces Marfanoid-progeroid-lipodystrophy (MPL) syndrome in rabbit. Disease models & mechanisms 2018, **11**(4).

9. Song Y, Xu Y, Liang M, Zhang Y, Chen M, Deng J, Li Z. CRISPR/Cas9-mediated mosaic mutation of SRY gene induces hermaphroditism in rabbits. Bioscience reports 2018, **38**(2).

10. Sui T, Liu D, Liu T, Deng J, Chen M, Xu Y, Song Y, Ouyang H, Lai L, Li Z. LMNA-mutated Rabbits: A Model of Premature Aging Syndrome with Muscular Dystrophy and Dilated Cardiomyopathy. Aging Dis 2019, **10**(1)**:** 102-115.

11. Liu H, Sui T, Liu D, Liu T, Chen M, Deng J, Xu Y, Li Z. Multiple homologous genes knockout (KO) by CRISPR/Cas9 system in rabbit. Gene 2018, **647:** 261-267.

12. Deng J, Chen M, Liu Z, Song Y, Sui T, Lai L, Li Z. The disrupted balance between hair follicles and sebaceous glands in Hoxc13-ablated rabbits. FASEB journal : official publication of the Federation of American Societies for Experimental Biology 2019, **33**(1)**:** 1226-1234.

13. Yao B, Liang M, Liu H, Sui T, Song Y, Zhang Y, Deng J, Xu Y, Lai L, Li Z. The minimal promoter (P1) of Xist is non-essential for X chromosome inactivation. RNA biology 2020, **17**(5)**:** 623-629.

14. Liu T, Wang J, Xie X, Wang K, Sui T, Liu D, Lai L, Zhao H, Li Z, Feng JQ. DMP1 Ablation in the Rabbit Results in Mineralization Defects and Abnormalities in Haversian Canal/Osteon Microarchitecture. Journal of bone and mineral research : the official journal of the American Society for Bone and Mineral Research 2019, **34**(6)**:** 1115-1128.

15. Song Y, Sui T, Zhang Y, Wang Y, Chen M, Deng J, Chai Z, Lai L, Li Z. Genetic deletion of a short fragment of glucokinase in rabbit by CRISPR/Cas9 leading to hyperglycemia and other typical features seen in MODY-2. Cellular and molecular life sciences : CMLS 2020, **77**(16)**:** 3265-3277.

16. Xu Y, Liu H, Pan H, Wang X, Zhang Y, Yao B, Li N, Lai L, Li Z. CRISPR/Cas9-mediated Disruption of Fibroblast Growth Factor 5 in Rabbits Results in a Systemic Long Hair Phenotype by Prolonging Anagen. Genes 2020, **11**(3).

17. Chen S, Xie W, Liu Z, Shan H, Chen M, Song Y, Yu H, Lai L, Li Z. CRISPR Start-Loss: A Novel and Practical Alternative for Gene Silencing through Base-Editing-Induced Start Codon Mutations. Molecular therapy Nucleic acids 2020, **21:** 1062-1073.

18. Liu Z, Chen M, Chen S, Deng J, Song Y, Lai L, Li Z. Highly efficient RNA-guided base editing in rabbit. Nature communications 2018, **9**(1)**:** 2717.

**Supplementary Figures**

**
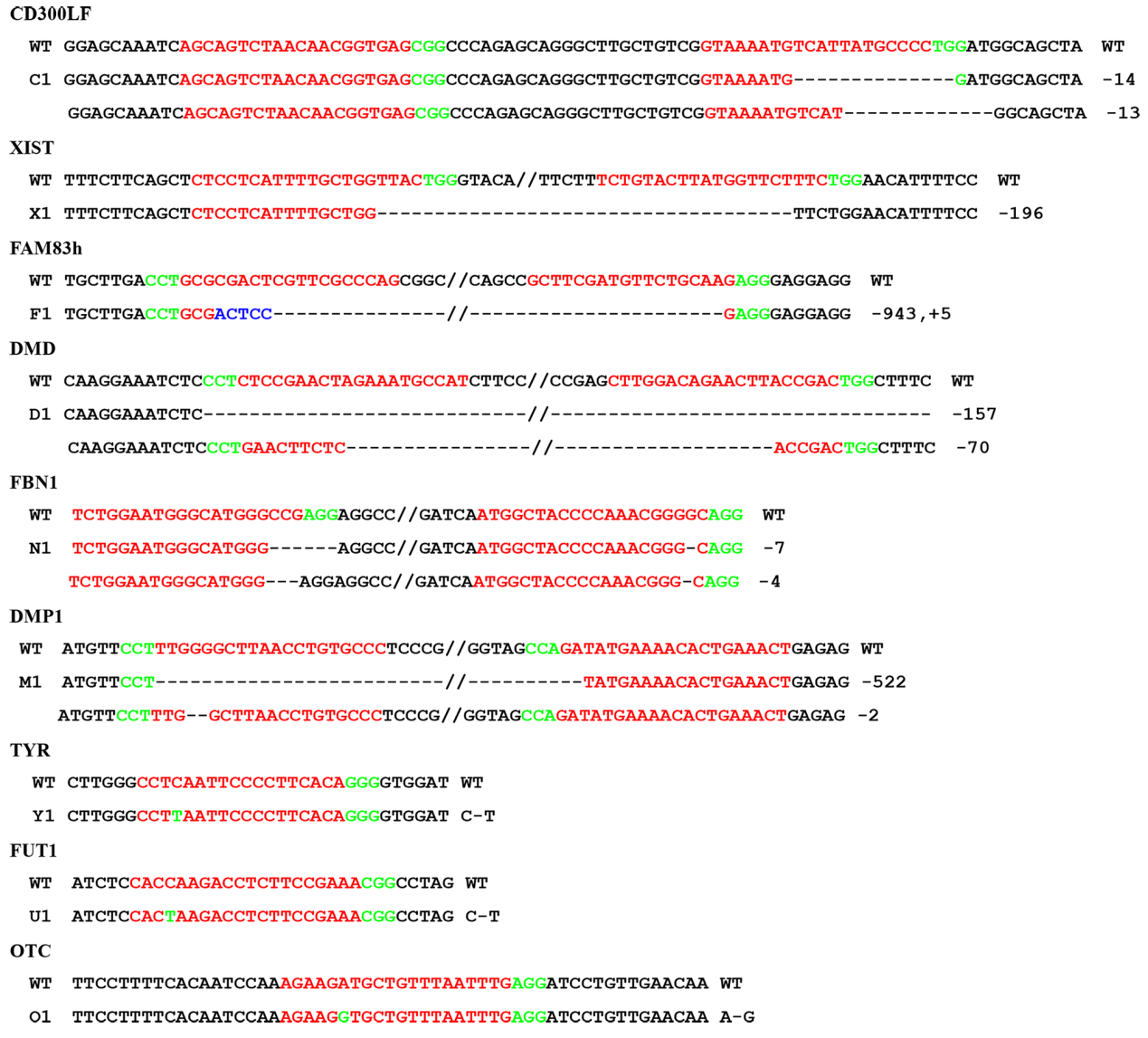
**

**Figure S1:** **Mutation detection of the 10 gene-edited rabbits by T-cloning and Sanger sequencing.**

T-cloning and Sanger sequencing of modified gene alleles in pups for sgRNA. WT sequence is shown at the top of the targeting sequence. PAM sites are highlighted in green; target sequences are shown in red; deletions (-); insertions are shown in blue; WT, wild-type control.


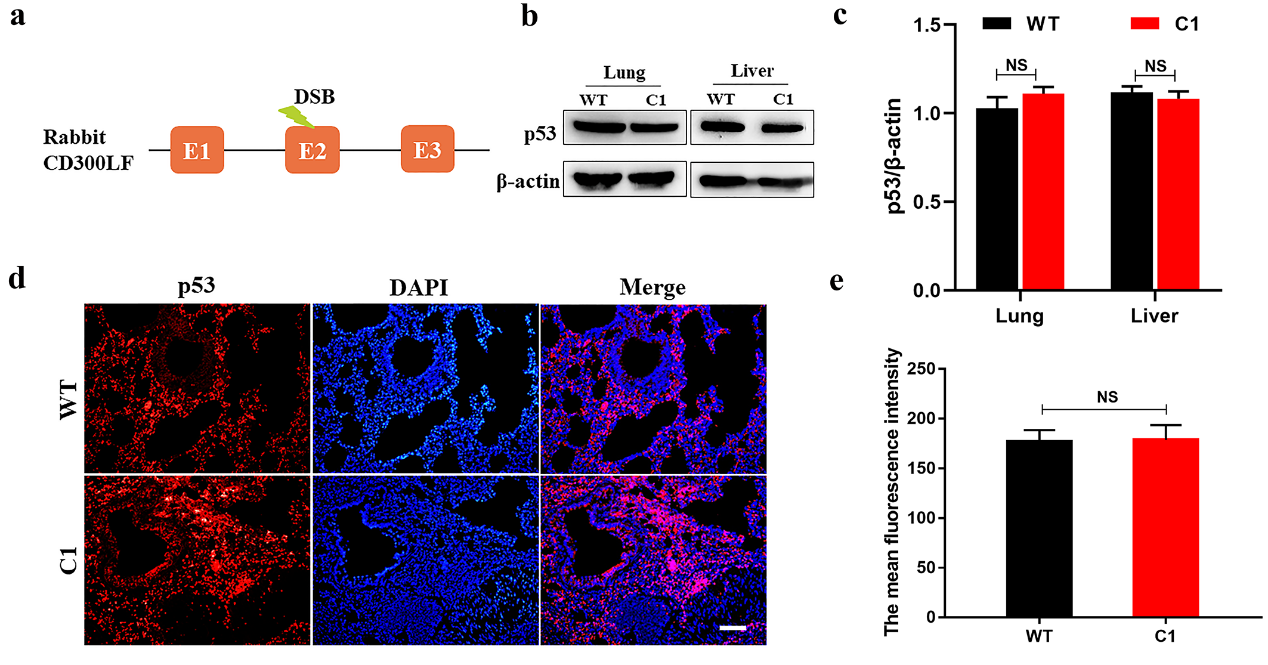


**Figure S2: The DSB generated in exon 2 of *CD300LF* gene not induces changes of p53 expression.**

(a) Schematic diagram of DSB generate sites in exon 2 of the rabbit *CD300LF* locus. *CD300LF* exons are indicated by the yellow boxes; the DSB is indicated by lightning shape. (b) Lung and liver protein levels of p53 and β-actin in rabbits. (c) Quantification of lung and liver protein levels of p53 and β-actin. (d) Fluorescence intensity and nuclear translocation of p53 in lung tissue of rabbits. (e) Quantification of Fluorescence intensity of p53. Scale bar: 50 µm, NS, no significant.


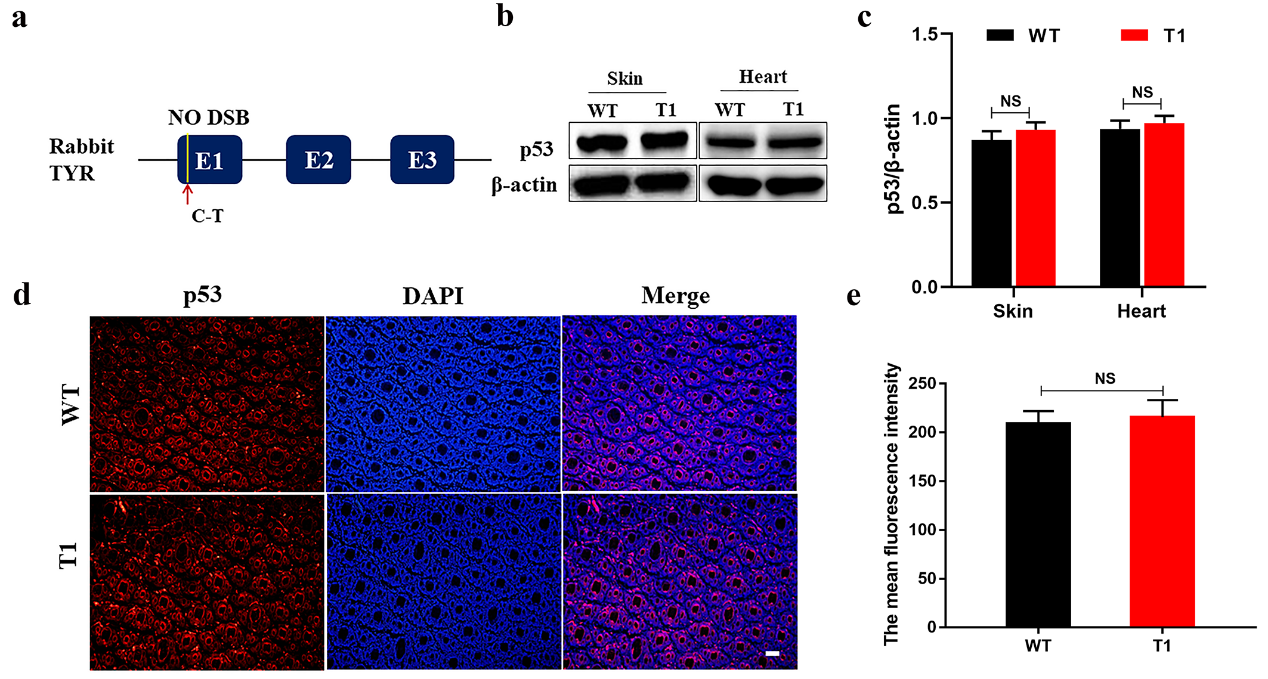


**Figure S3: The C>T base mutation in exon 1 of *TYR* gene not causes changes of p53 expression.**

(a) Schematic diagram of single base mutation generates sites in exon 1 of the rabbit *TYR* locus. *TYR* exons are indicated by the blue boxes. (b) Skin and heart protein levels of p53 and β-actin in rabbits. (c) Quantification of skin and heart protein levels of p53 and β-actin. (d) Fluorescence intensity and nuclear translocation of p53 in skin tissue of rabbits. (e) Quantification of Fluorescence intensity of p53. Scale bar: 50 µm, NS, no significant.


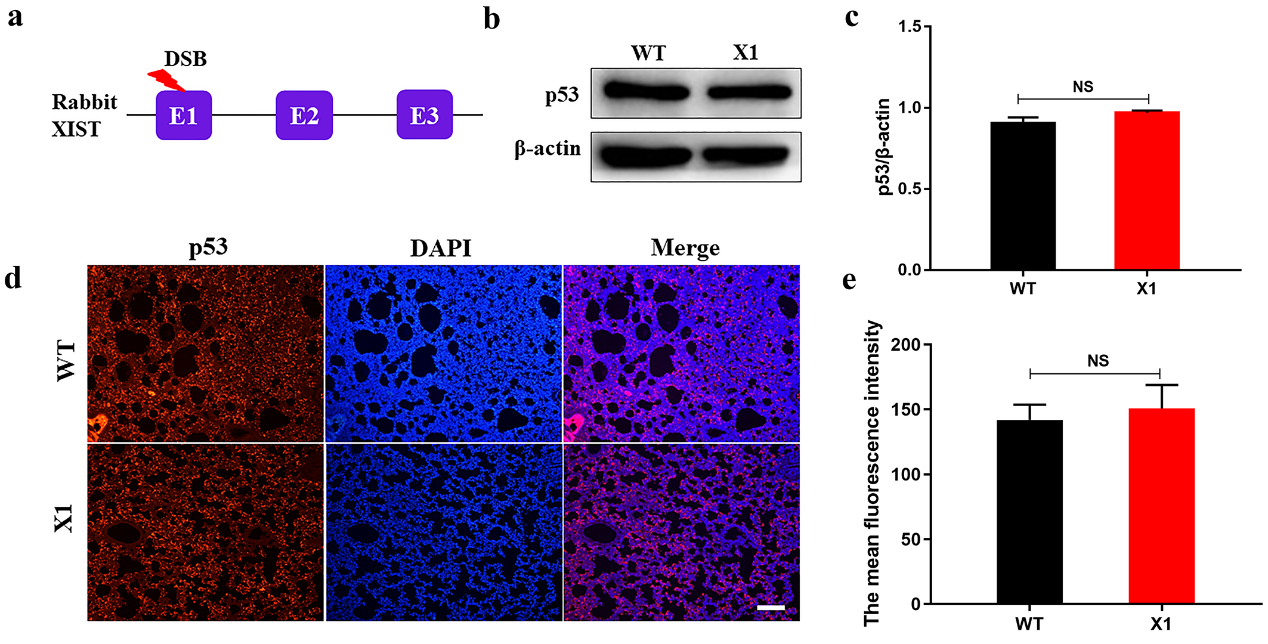


**Figure S4: The DSB generated in exon 1 of *XIST* gene not induces changes of p53 expression.**

(a) Schematic diagram of DSB generate sites in exon 1 of the rabbit *XIST* locus. *XIST* exons are indicated by the purple boxes; the DSB is indicated by lightning shape. (b) Lung protein levels of p53 and β-actin in rabbits. (c) Quantification of lung protein levels of p53 and β-actin. (d) Fluorescence intensity and nuclear translocation of p53 in lung tissue of rabbits. (e) Quantification of Fluorescence intensity of p53. Scale bar: 50 µm, NS, no significant.


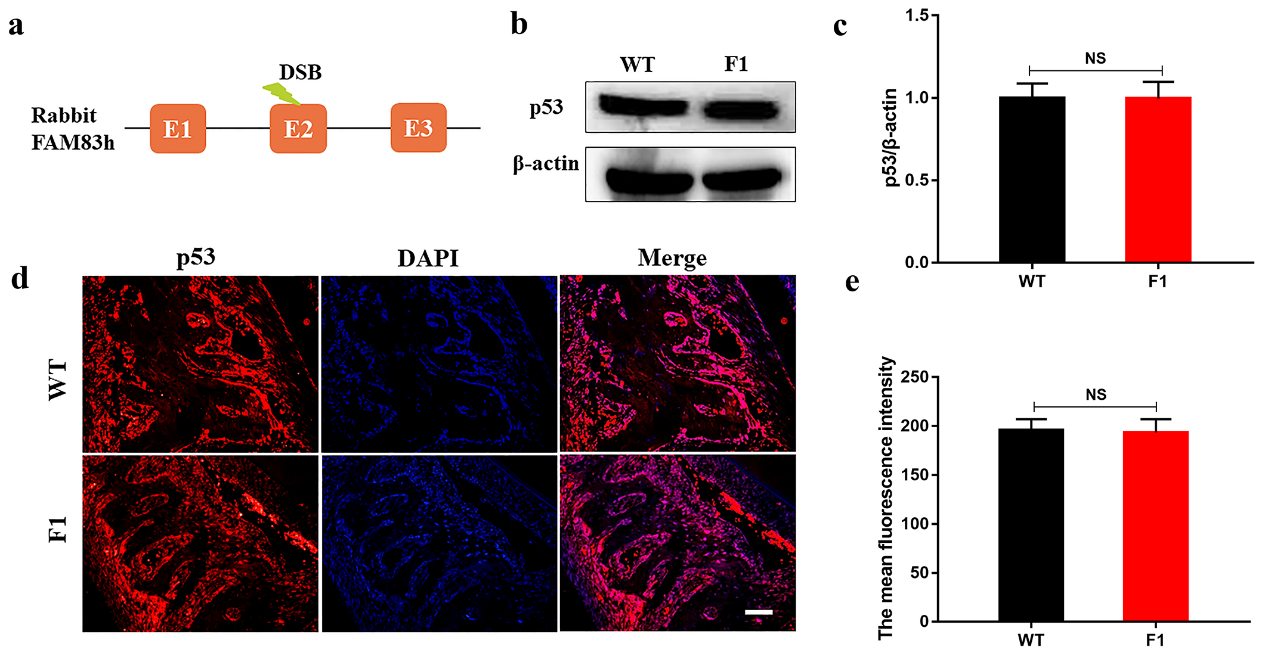


**Figure S5: The DSB generated in exon 1 of *FAM83h* gene not induces changes of p53 expression.**

(a) Schematic diagram of DSB generate sites in exon 1 of the rabbit *FAM83h* locus. *FAM83h* exons are indicated by the yellow boxes; the DSB is indicated by lightning shape. (b) Bone protein levels of p53 and β-actin in rabbits. (c) Quantification of bone protein levels of p53 and β-actin. (d) Fluorescence intensity and nuclear translocation of p53 in bone tissue of rabbits. (e) Quantification of Fluorescence intensity of p53. Scale bar: 50 µm, NS, no significant.


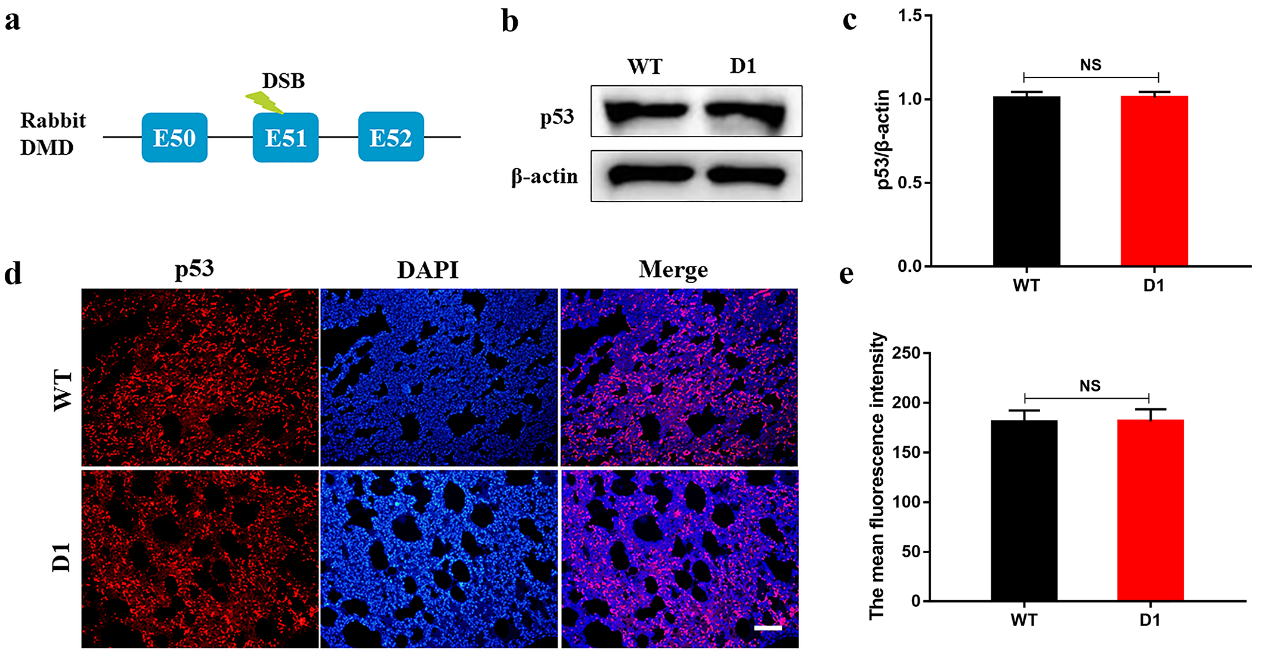


**Figure S6: The DSB generated in exon 51 of *DMD* gene not induces changes of p53 expression.**

(a) Schematic diagram of DSB generate sites in exon 51 of the rabbit *DMD* locus. *DMD* exons are indicated by the blue boxes; the DSB is indicated by lightning shape. (b) Lung protein levels of p53 and β-actin in rabbits. (c) Quantification of lung protein levels of p53 and β-actin. (d) Fluorescence intensity and nuclear translocation of p53 in lung tissue of rabbits. (e) Quantification of Fluorescence intensity of p53. Scale bar: 50 µm, NS, no significant.


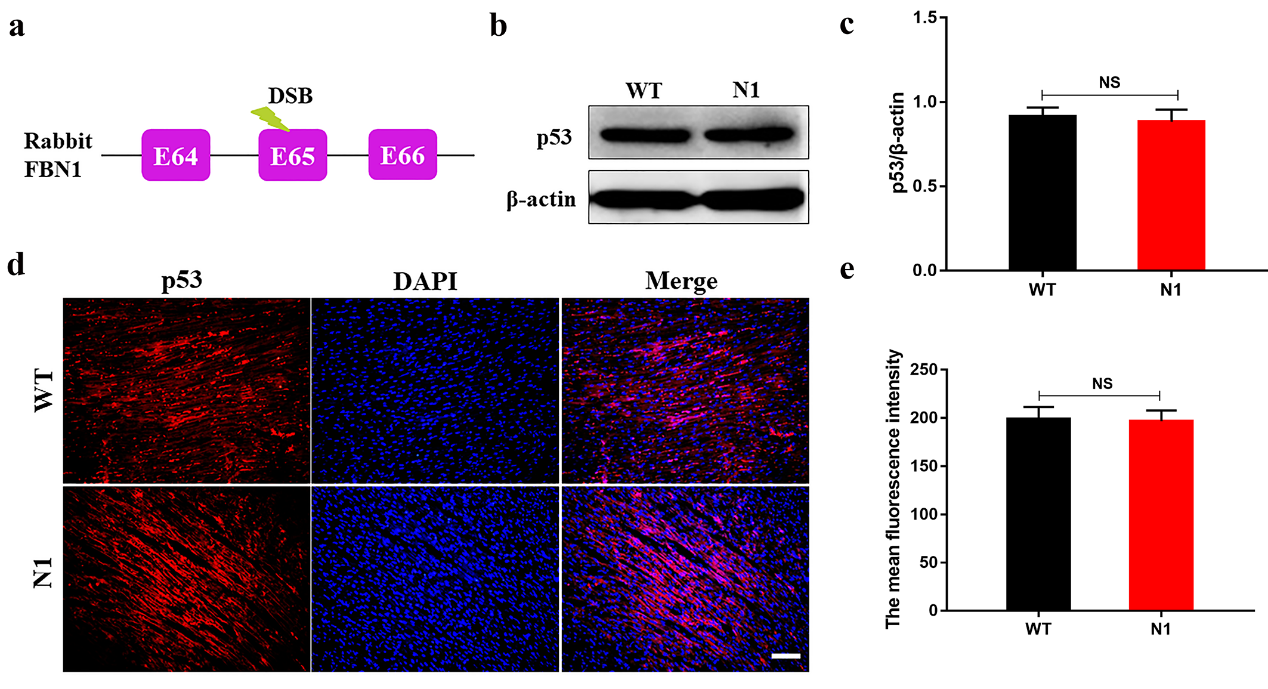


**Figure S7: The DSB generated in exon 65 of *FBN1* gene not induces changes of p53 expression.**

(a) Schematic diagram of DSB generate sites in exon 65 of the rabbit *FBN1* locus. *FBN1* exons are indicated by the purple boxes; the DSB is indicated by lightning shape. (b) Cardiac muscle protein levels of p53 and β-actin in rabbits. (c) Quantification of cardiac muscle protein levels of p53 and β-actin. (d) Fluorescence intensity and nuclear translocation of p53 in cardiac muscle tissue of rabbits. (e) Quantification of Fluorescence intensity of p53. Scale bar: 50 µm, NS, no significant.


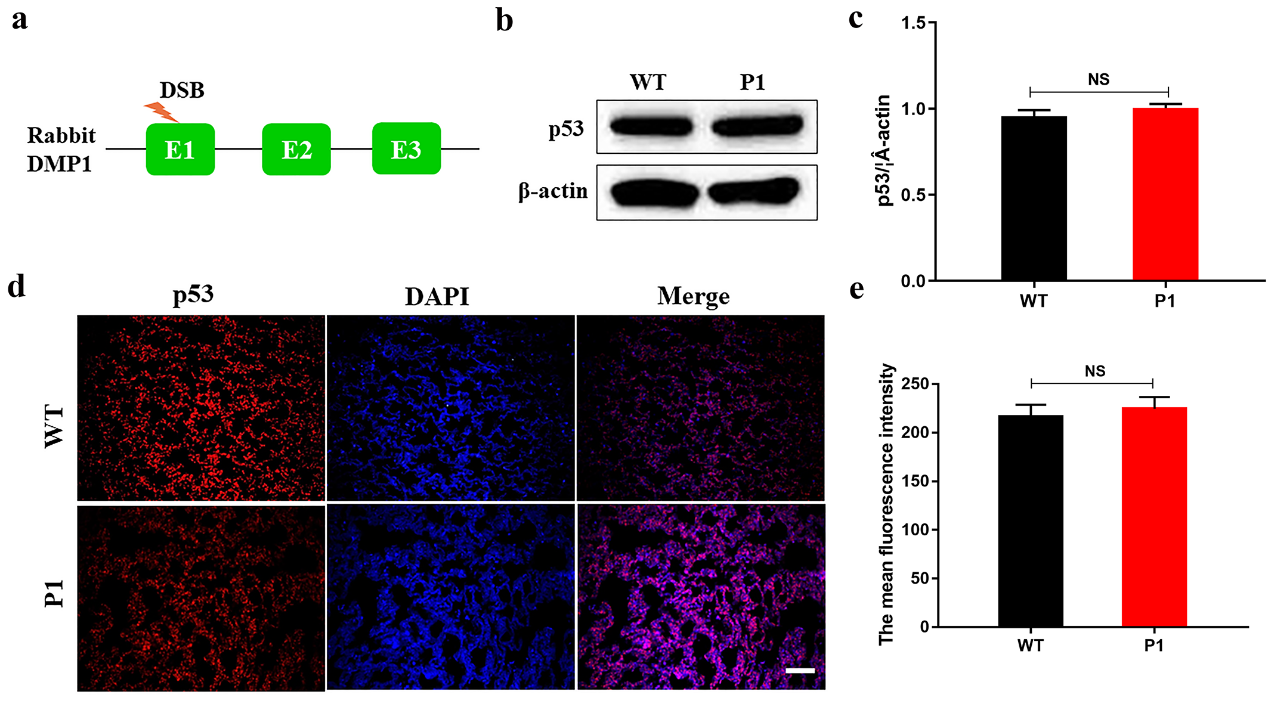


**Figure S8: The DSB generated in exon 1 of *DMP1* gene not induces changes of p53 expression.**

(a) Schematic diagram of DSB generate sites in exon 1 of the rabbit *DMP1* locus. *DMP1* exons are indicated by the green boxes; the DSB is indicated by lightning shape. (b) Lung protein levels of p53 and β-actin in rabbits. (c) Quantification of lung protein levels of p53 and β-actin. (d) Fluorescence intensity and nuclear translocation of p53 in lung tissue of rabbits. (e) Quantification of Fluorescence intensity of p53. Scale bar: 50 µm, NS, no significant.


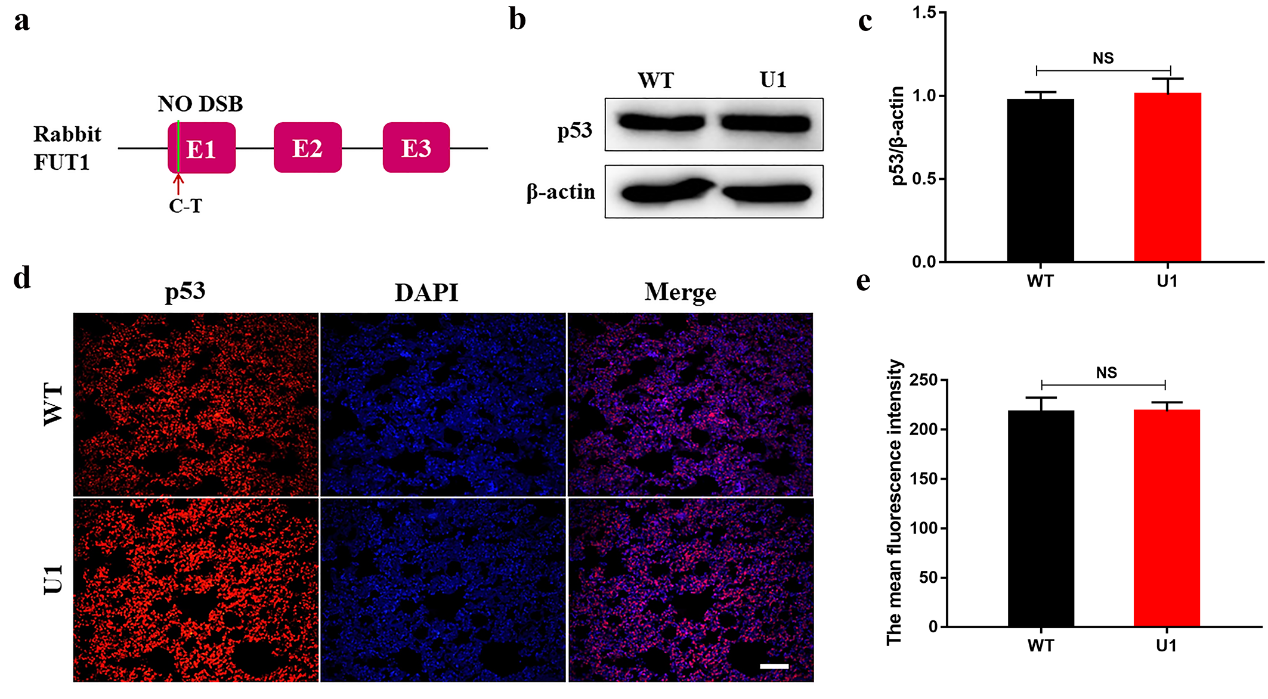


**Figure S9: The C>T base mutation in exon 1 of *FUT1* gene not induces changes of p53 expression.**

(a)Schematic diagram of single base mutation generate sites in exon 1 of the rabbit *FUT1* locus. *FUT1* exons are indicated by the pink boxes. (b) Lung protein levels of p53 and β-actin in rabbits. (c) Quantification of lung protein levels of p53 and β-actin. (d) Fluorescence intensity and nuclear translocation of p53 in lung tissue of rabbits. (e) Quantification of Fluorescence intensity of p53. Scale bar: 50 µm, NS, no significant.


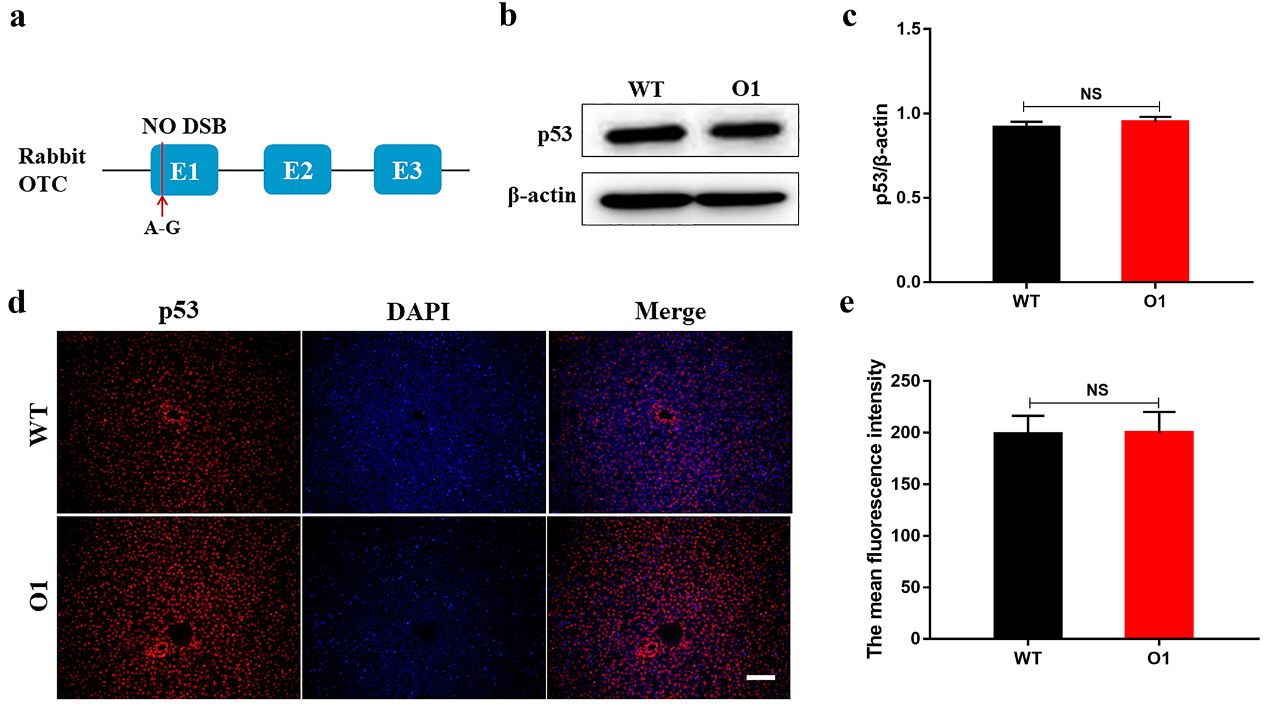


**Figure S10: The A>G base mutation in exon 1 of *OTC* gene not induces changes of p53 expression.**

(a) Schematic diagram of single base mutation generate sites in exon 1 of the rabbit *OTC* locus. *OTC* exons are indicated by the blue boxes. (b) Liver protein levels of p53 and β-actin in rabbits. (c) Quantification of liver protein levels of p53 and β-actin. (d) Fluorescence intensity and nuclear translocation of p53 in liver tissue of rabbits. (e) Quantification of Fluorescence intensity of p53. Scale bar: 50 µm, NS, no significant.
